# Surgical abscess enolization for complex mitroaortic endocarditis treatment in a high risk patient

**DOI:** 10.1186/1749-8090-8-S1-O299

**Published:** 2013-09-11

**Authors:** I Moriones, J Berjon, J Fernandez, R Sanchez, L Jimenez, F Gomez

**Affiliations:** 1 Department of Cardiac Surgery, Complejo Hospitalario de Navarra, Pamplona, Spain

## 

We know that the mitroaortic endocarditis is one of the worse and risky surgical situation, particularly when it happens in patients previously operated and whether is involved the mitroaortic continuity and atrial septum. In this paper we describe here the surgical solution applied to one patient.

We present here a 79-year old man with symptoms of fever, leukocytosis, confusional state, vomits, artralgia, A-V blockage with transient pacemaker implantation, renal insufficiency and peripheral embolization treated by embolectmy. His medical history included aortic valve replacement, two years before, by Mitroflow biological prosthesis, diabetes, and asymptomatic prostatic carcinoma. Blood cultures detected streptococcus bovis. Transeophageal echocardiography showed images of endocarditis of the aortic prosthesis with one abscess in the posterolateral aortic annulus that extends in to the the atrial septum and the anterior mitral annulus with suspected vegetations and mitral mild regurgitation.

The patient was treated with levofloxacin, gentamycine, vancomycin and ceftriaxone. After 20 days of conservative therapy, the patient underwent surgery.

A median sternotomy and standard cannulation were performed Moderate hypothermia and antegrade blood cardioplegia were administered. After the opening of the aorta, previous aortic biological prosthesis was extracted and the abscess affecting posterolateral part of the ring was cleaned without extraction of solid material of the rest of the abscess. After this we injected 20 cc of ethylic alcohol for embolism the abscess and for tanning the affected aortic annulus. Previous Aortic prosthesis was replaced by a mechanical 20- mm ATS (ATS Medical, Inc.Minneapolis USA), then we opened the left atrium and proceeded to remove the vegetation inserted into the anterior annulus After this, injected again 10 cc of alcohol in the abscess from the left atrium until the liquid appeared on the aortic face of the abscess .In the postoperative course during the initial 24 hours presented low cardiac output and needed inotropic support and intraortic balloon pump assistance. The patient was weaned from respirator machine five days later with a good recovery. Permanent pacemaker was implanted twelve days later. The patients was discharged from the hospital 48 days later after surgery, without fever had recovered renal function and good clinical situation Predischarge echocardiography, one month later, showed excellent function of the prosthesis and one posterior residual cavity in the outflow tract of left ventricle corresponding to previous abscess zone. Mitral valve function was successful without regurgitation. Ejection fraction was 0.4

## Comment

Is generally accepted that endocarditis is a very important risk factor, particularly in older patients and in the cases operated before with cardiac valve prosthesis. In this particular patient, the extension of the infection affecting aortic and mitral valves, the intervalvular fibrous body and interatrial septum associated to A-V blockage associated to the important comorbidity, was a very important challenge from the surgical point of view. For this reason we decided to realize described intervention to reduce potentially the surgical risk and to save the patient life. We used total etilic alcohol, knowing bacteriostatic properties of this liquid and for tanning and reduce friability valve ring and the other cardiac tissues. In this way we intended to reduce the risk and complications of the monoblock technique making a simpler operation it was not necessary to replace the mitral valve, performing only extraction of vegetation and new injection of alcohol in the abscess from the mitral side.

## Conclusion

The good result obtained in this patient, leads us to consider the possibility of using this method in other similar patients.

